# Importance of hospital and clinical factors for early mortality in Takotsubo syndrome: Insights from the Swedish Coronary Angiography and Angioplasty Registry

**DOI:** 10.1186/s12872-024-04023-6

**Published:** 2024-07-15

**Authors:** Thorsteinn Gudmundsson, Björn Redfors, Truls Råmunddal, Oskar Angerås, Petur Petursson, Araz Rawshani, Henrik Hagström, Joakim Alfredsson, Christina Ekenbäck, Loghman Henareh, Kristofer Skoglund, Charlotta Ljungman, Moman Mohammad, Tomas Jernberg, Ole Fröbert, David Erlinge, Elmir Omerovic

**Affiliations:** 1 Department of Cardiology, Department of Molecular and Clinical Medicine, Sahlgrenska University Hospital Institute of Medicine, Sahlgrenska Academy at University of Gothenburg, Bruna stråket 16, Gothenburg, 41345 Sweden; 2 Department of Cardiology, Umeå University Hospital, Umeå, Sweden; 3 Department of Cardiology, University Hospital, Linköping, Sweden; 4 Department of Cardiology, Danderyd University Hospital, Stockholm, Sweden; 5 Department of Cardiology, Karolinska University Hospital, Stockholm, Sweden; 6 Department of Cardiology, Skåne University Hospital, Lund, Sweden; 7 Department of Cardiology, Örebro University Hospital, Örebro, Sweden

**Keywords:** Takotsubo syndrome, 30-day mortality, Machine learning, Predictors of mortality, Swedish Coronary Angiography and Angioplasty Registry (SCAAR), Gradient boosting

## Abstract

**Background:**

Takotsubo syndrome (TTS) is an acute heart failure syndrome with symptoms similar to acute myocardial infarction. TTS is often triggered by acute emotional or physical stress and is a significant cause of morbidity and mortality. Predictors of mortality in patients with TS are not well understood, and there is a need to identify high-risk patients and tailor treatment accordingly. This study aimed to assess the importance of various clinical factors in predicting 30-day mortality in TTS patients using a machine learning algorithm.

**Methods:**

We analyzed data from the nationwide Swedish Coronary Angiography and Angioplasty Registry (SCAAR) for all patients with TTS in Sweden between 2015 and 2022. Gradient boosting was used to assess the relative importance of variables in predicting 30-day mortality in TTS patients.

**Results:**

Of 3,180 patients hospitalized with TTS, 76.0% were women. The median age was 71.0 years (interquartile range 62–77). The crude all-cause mortality rate was 3.2% at 30 days. Machine learning algorithms by gradient boosting identified treating hospitals as the most important predictor of 30-day mortality. This factor was followed in significance by the clinical indication for angiography, creatinine level, Killip class, and age. Other less important factors included weight, height, and certain medical conditions such as hyperlipidemia and smoking status.

**Conclusions:**

Using machine learning with gradient boosting, we analyzed all Swedish patients diagnosed with TTS over seven years and found that the treating hospital was the most significant predictor of 30-day mortality.

**Supplementary Information:**

The online version contains supplementary material available at 10.1186/s12872-024-04023-6.

## Introduction

Takotsubo syndrome (TTS), also known as stress cardiomyopathy or broken heart syndrome, is an acute cardiac condition that clinically resembles an acute myocardial infarction [1, 2]. Characterized by transient left ventricular dysfunction, TTS often occurs in response to severe emotional or physical stressors [3]. While the left ventricular dysfunction observed in TTS typically exhibits a transient nature, complete recovery does not happen in all cases, with some patients experiencing prolonged or permanent effects [4]. Despite the transient nature of ventricular dysfunction, TTS has been recognized as a significant contributor to morbidity and mortality [5, 6].

While the exact pathophysiology of TTS remains under investigation, it is thought to involve a surge in catecholamines leading to myocardial stunning [3, 7]. Epidemiologically, the syndrome has a predilection for postmenopausal women, although it can affect individuals of any age or sex [1, 2]. The overall incidence of TTS seems to be increasing, potentially due to heightened awareness and improved recognition of the condition [6]. Despite the growing body of literature on TTS, predictors of mortality, especially short-term mortality, remain inadequately understood. Understanding these predictors is paramount in clinical practice as it would allow for risk stratification and individualized treatment plans, potentially improving patient outcomes. Recent advances in machine learning provide a robust platform to explore complex interrelations among clinical variables [8–10]. Cardiology has increasingly used this approach to predict outcomes and guide treatment strategies, showcasing a promising avenue for understanding and managing complex conditions like TTS [11–14].

Against this background, we aimed in the present study to leverage machine learning techniques to evaluate potential predictors of 30-day mortality in TTS patients. Using data from the nationwide Swedish Coronary Angiography and Angioplasty Registry (SCAAR), we examined a comprehensive range of clinical factors and their relative importance in predicting short-term outcomes.

## Methods

### Study population and data collection

Our study population comprised all patients diagnosed with TTS between 2015 and 2022, as identified in SCAAR. The nationwide registry captures all coronary angiographies and angioplasties performed in Sweden. The interventional cardiologist registers TTS cases in SCAAR using criteria outlined in Table 1, which is consistent with the current standards of the European Society of Cardiology [5]. There are 31 hospitals in Sweden that perform coronary angiography and report it to the SCAAR registry. These are secondary or tertiary hospitals, including nine university hospitals, serving a population ranging from 50,000 to 1.000.000 inhabitants. The Swedish Ethical Review Authority reviewed and approved the study (Diarie nr. 759 − 13 and 2022–03103). The need to obtain individual informed consent to participate was waived.

**Table 1:** Definition of takotsubo syndrome used in the Swedish coronary angiography and angioplasty registry. The criteria are based on the gothenburg criteria [15, 16]

1. Transient wall motion abnormalities in the left or right ventricle (preceding negative emotional stress or somatic disease, trauma etc.) is often, but not always, present
2. Absence of angiographic signs of plaque rupture (note that significant coronary artery disease may coexist with takotsubo syndrome)
3. New ECG changes (ST-elevation or ST depression or T-wave inversion)
4. Absence of myocarditis.

### Outcome measures

We defined the primary outcome measure as 30-day all-cause mortality following hospitalization for TTS, encompassing any cause of death within 30 days from the initial hospital admission for TTS. Data about mortality was missing for 58 patients; 54 had foreign social security numbers, and information on four patients was not updated from the national death registry to SCAAR.

### Predictor variables and outcome

We considered a range of candidate predictors, including demographic characteristics, clinical presentation for angiography (suspected ACS, stable angina, arrhythmia, heart failure, or other), hemodynamic parameters, biomarkers, clinical history, and the hospital where the patient was treated (Table 2). We chose these variables for their potential clinical relevance and impact on outcomes in TTS patients.

**Table 2:** Demographic and Clinical Characteristics of Takotsubo Syndrome Patients

Patient Characteristics	*N* = 3,180^*1*^
Female sex	2,422 (76%)
Age, years	71 (62, 77)
Presentation	
Acute	2809 (88.3%)
Not acute	371 (11.7%)
BMI	25.4 (22.6, 28.9)
Height, cm	167 (162, 173)
Weight, kg	71 (61, 83)
Creatinine, mmol/L	71 (59, 85)
Diabetes	466 (15%)
Smoking	1,083 (34%)
Hypertension*	1,781 (56%)
Hyperlipidemia#	1,073 (34%)
Previous MI	373 (12%)
Previous stroke	61 (6.7%)
PAD	27 (3.0%)
Angiography findings	
Normal	2,425 (76.3%)
SVD	372 (11.7%)
MVD	383 (12%)
Hospital type	
University Hospital	1549 (48.7)
Non-University hospital	1631 (51.3)
Year	
2015	463 (15%)
2016	514 (16%)
2017	390 (12%)
2018	378 (12%)
2019	376 (12%)
2020	358 (11%)
2021	361 (11%)
2022	333 (10%)
2023	7 (0.2%)
Mechanical assist device	
IABP	3 (0.09)
Impella	1 (0.03)
ECMO	1 (0.03)
Indication for angiography	
Stable angina	135 (4.2)
ACS	2413 (75.9)
Heart failure	202 (6.3)
Arrhythmia	34 (1.1)
Other	396 (12.5)
Killip class	
Killip 1	2,663 (91%)
Killip 2	170 (5.8%)
Killip 3	62 (2.1%)
Killip 4	36 (1.2%)

^*1*^ n (%); Median (inter quartile range)

BMI body mass index, PAD peripheral artery disease, SVD single vessel disease, MVD multivessel disease, ACS acute coronary syndrome (unstable angina, ST-elevation myocardial infarction, non-ST elevation myocardial infarction)

* Refers to patients with a prior diagnosis of hypertension who are currently receiving medication specifically for hypertension

# Refers to patients with a prior diagnosis of hyperlipidemia who are currently receiving medication specifically for hyperlipidaemia

### Statistical analysis and machine learning

#### Pre-processing

Missing values in the dataset were imputed using the missRanger package, which utilizes the random forest algorithm to fill in missing values [17, 18]. The missRanger method has been shown to produce accurate imputations while preserving the data distribution [19]. The continuous variables were normalized to ensure they were on the same scale.

#### Modeling strategy

We used a gradient-boosting machine learning algorithm to (1) calculate relative variable importance, analyze the data [17, 20] and (2) derive a prediction model for survival at 30 days [18]. Gradient boosting is a machine learning algorithm with a tree-based ensemble created using gradient descent. This algorithm builds an additive model in a forward stage-wise fashion, allowing for the optimization of arbitrary differentiable loss functions. In our case, we used logistic loss, which is appropriate for binary outcomes like 30-day mortality. We assessed the relative importance of each variable in predicting 30-day mortality. This approach allows the model to identify complex, non-linear relationships and interactions between predictor variables that traditional statistical methods may miss.

#### Cross-validation

We split the original dataset into training (70%) and test (30%) sets. Model development was performed solely on the training set. We stratified the train-test-split on the outcome to preserve the outcome’s prevalence in the train and test sets. Hyperparameter tuning was performed using an automatic grid search. We tested various models with varying hyperparameters to identify the optimal configuration. Specifically, the hyperparameter grid included a range of shrinkage values (learning rates) set at 0.01, 0.1, and 0.3, interaction depths of 1, 3, and 5, minimum observations in a node set at 5, 10, and 15, and bag fractions (subsample ratios) of 0.65, 0.8, and 1. We did not specify a fixed number of trees in the grid, allowing the model to determine the optimal number during training. To select the best model, we employed 5-fold cross-validation, repeated five times on the training set, focusing on optimizing the ROC AUC as our performance metric.

#### Final evaluation

The final model evaluation was done by testing the final model on the test set. We chose ROC AUC, F1, precision, and recall as metrics to evaluate the model. We did not compute uncertainty estimates for these statistics since the purpose of the study was primarily to explore the predictors and their general ability to predict the outcome. We performed internal validation of our model using a bootstrap approach. A bootstrap sample set was drawn from our original dataset, and the gradient boosting model was applied to each. The model’s performance was then evaluated on the original dataset, providing an unbiased estimate of the model’s predictive ability. All statistical analyses were performed using R statistical software (version 4.3.2). The ‘gbm’ package (version 2.1.8.1) was used for the gradient boosting analysis.

## Results

### Patient characteristics

Our analysis included 3,180 patients; 76% were females, and the median age was 71 years (IQR 62–77). 56% had hypertension, and 34% had hyperlipidemia. A history of myocardial infarction was reported in 12%, and 6,7% of the subjects had a previous stroke. Peripheral artery disease was present in only 3% of the study population [21, 22]. In terms of the severity of coronary artery disease, 76,3% had no significant stenosis, 11,7% had single-vessel disease, and 12% had multivessel disease. There was an even distribution of patients across the years. The most common indication for coronary angiography was acute coronary syndrome, with less than 5% performed for stable angina. Killip class 1 was the most common, encompassing more than 90% of the cases. Kidney failure occurred in 2 (0.06%) patients after coronary angiography.

**Fig. 1:**
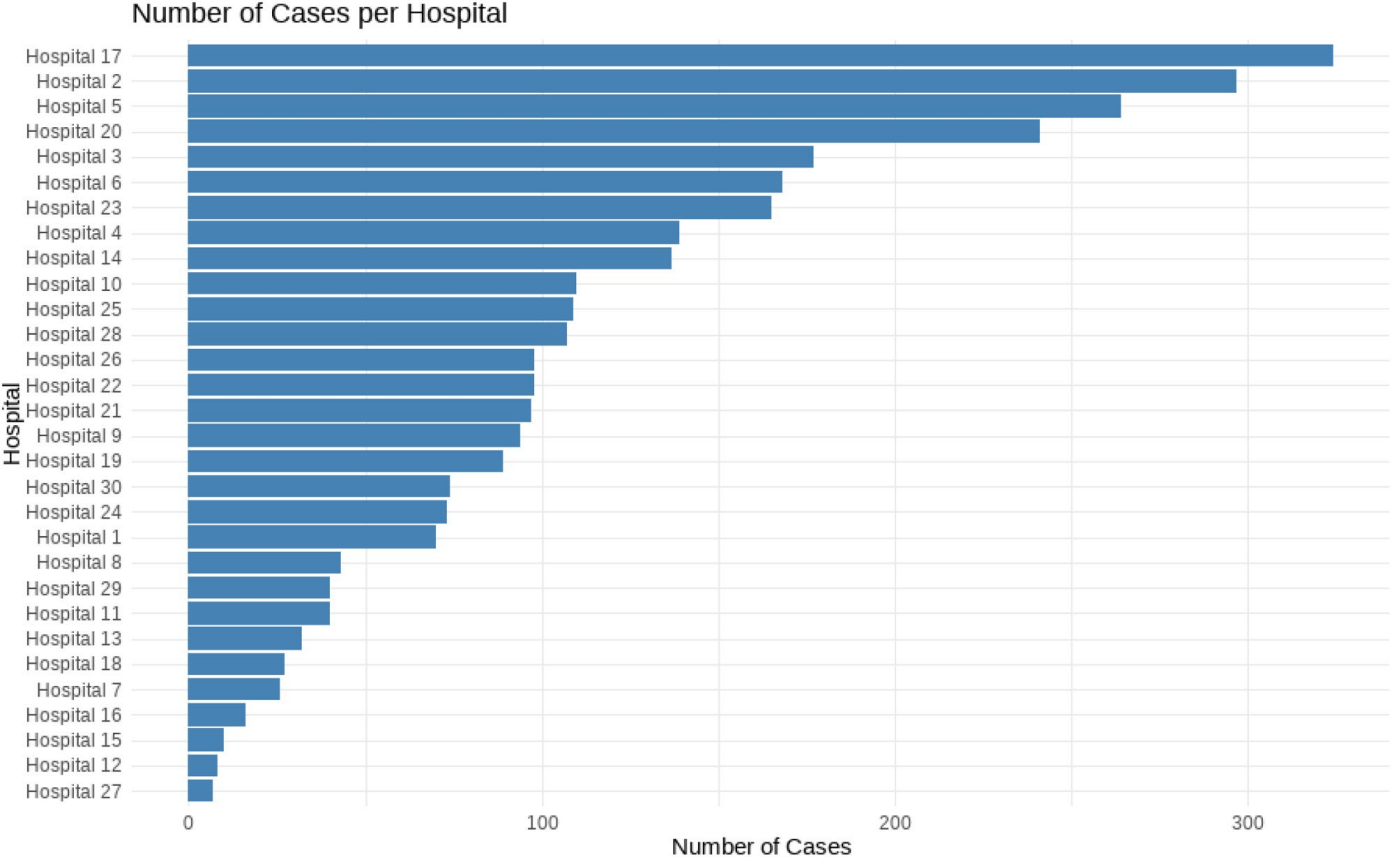
“Number of Takotsubo Syndrome Cases per Hospital” - This bar chart illustrates the distribution of Takotsubo syndrome cases across various hospitals. Each bar represents a different hospital, with the height of the bar indicating the number of cases at that hospital. The hospitals are ordered from lowest to highest based on the number of reported cases

**Fig. 2:**
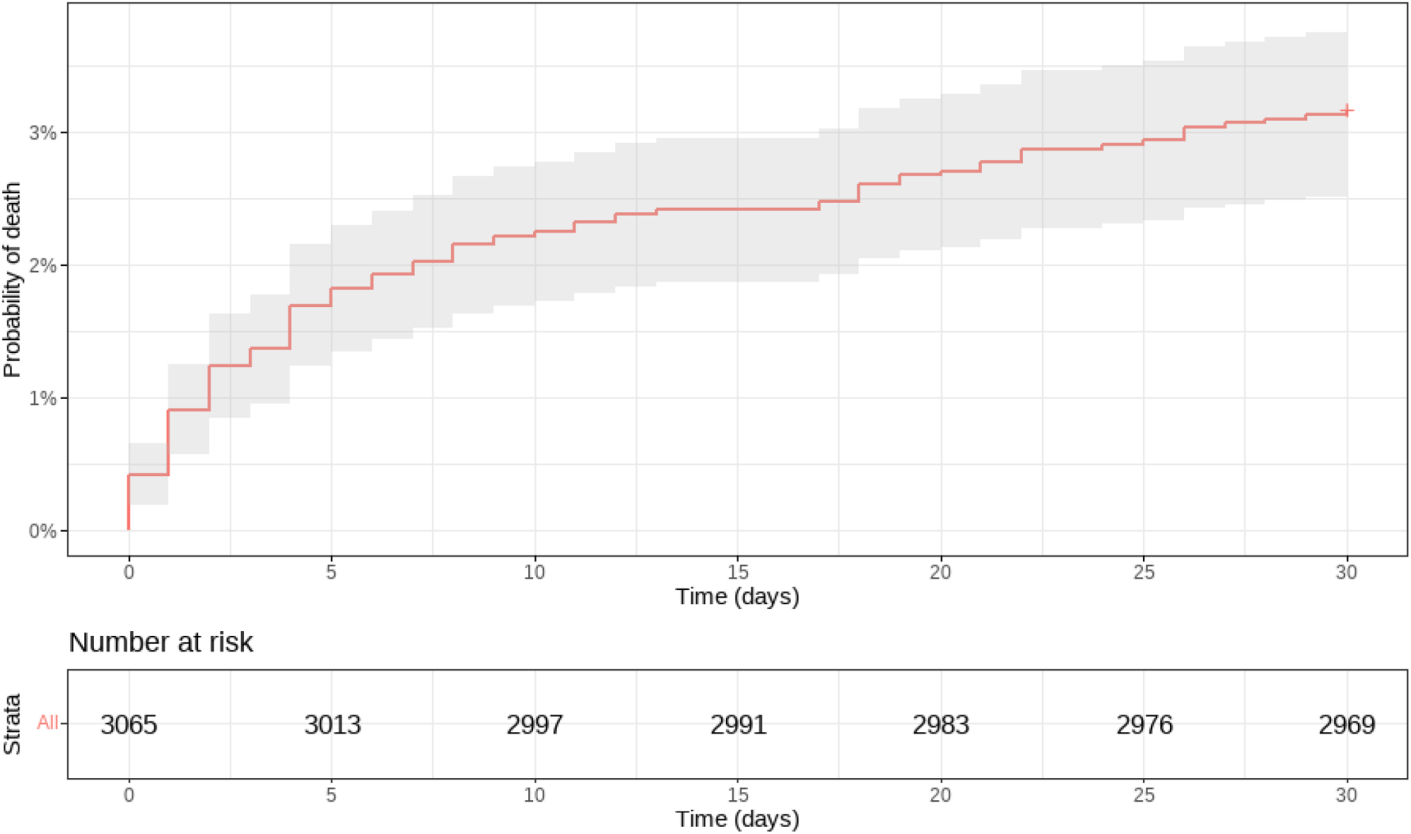
“30-Day Kaplan-Meier Mortality Curve for Takotsubo Syndrome” - This survival curve represents the estimated probability of surviving past 30 days for patients diagnosed with Takotsubo syndrome. The shaded area around the curve denotes the confidence interval. The graph also includes a risk table, showing the number of patients at risk at different time points. The estimated 30-day mortality rate for the population under study is 3.17%

**Fig. 3:**
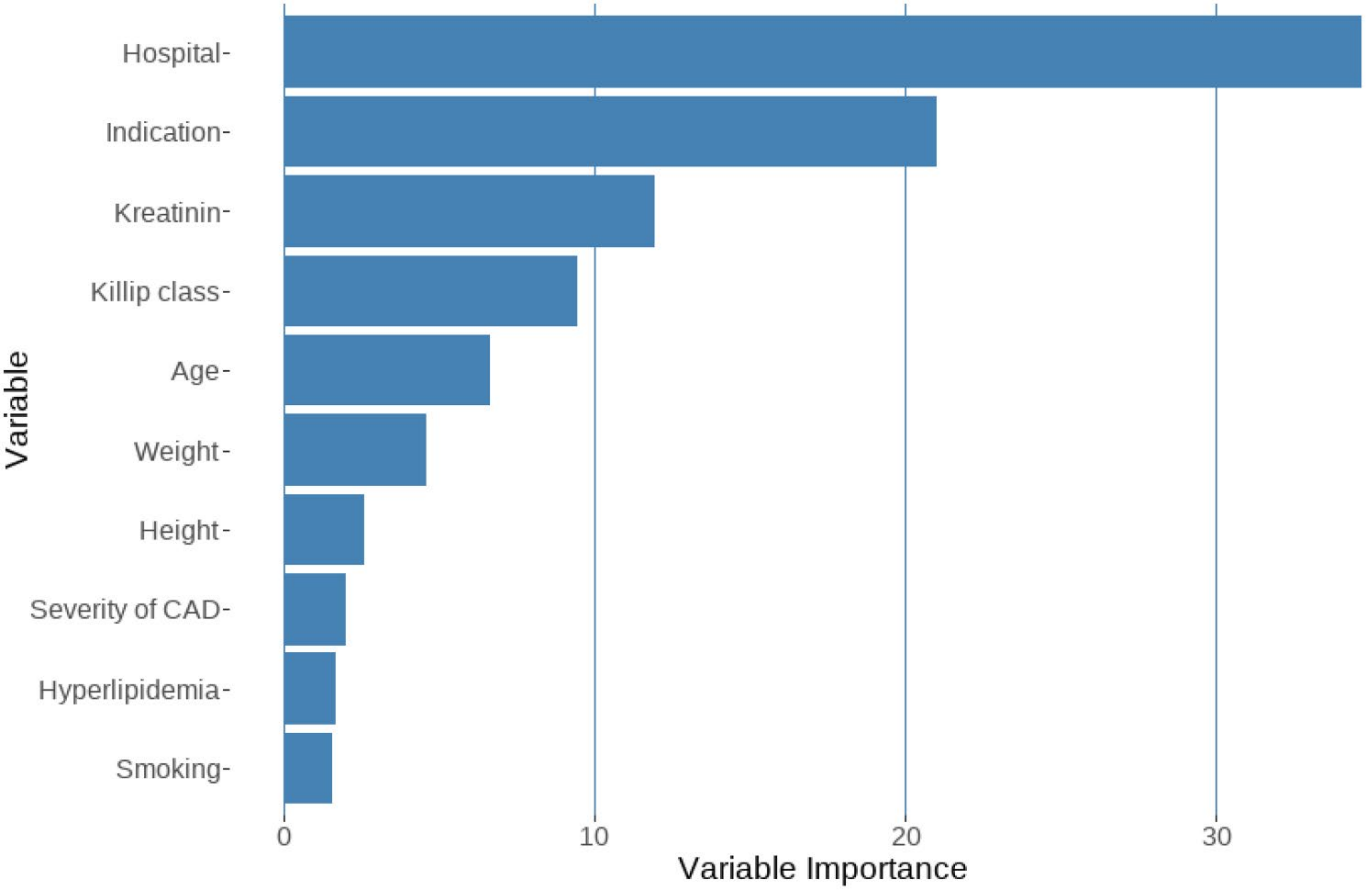
“Variable Importance for Predicting 30-Day Mortality in Takotsubo Syndrome” - This graph depicts the relative importance of different predictor variables included in the gradient boosting machine model for predicting 30-day mortality in patients with Takotsubo syndrome. Each variable is listed along the y-axis, and its importance is represented by the length of the bar on the x-axis

Reported cases per hospital varied, with numbers ranging from 7 to 324, as shown in Fig. 1. Thirty days after hospital admission, the crude all-cause mortality rate stood at 3.2%, depicted in Fig. 2. A gradient-boosting algorithm pinpointed a range of predictors linked to 30-day mortality. Figure 3 presents these predicting variables along with their respective explanatory power compared to each other.

### Gradient boosting machine model

The gradient boosting machine model was evaluated using various metrics and showed reliable performance. The accuracy was 96.8%, indicating most predictions were correct. Precision was 97.0%, reflecting the model’s effectiveness in correctly identifying positive cases. The recall was 99.8%, meaning the model captured nearly all positive cases. The F1 score was 98.4%, suggesting a well-balanced precision and recall. The area under the curve was also 86.9%, indicating a strong ability to differentiate the survival status.

The most important predictor of 30-day mortality was the hospital where the patient was treated, which accounted for 35.5% of the relative importance in the model. Clinical presentation at angiography was the second most important factor, contributing 21.1% to the model’s relative importance. Creatinine level and Killip class also provided significant information, accounting for 11.9% and 8.9% of the model’s relative importance, respectively. Age at angiography contributed 6.5% to the model’s relative importance, whereas factors like weight, height, hyperlipidemia, smoking status, and hypertension were less important. Gender and history of stroke had a minimal impact on predicting 30-day mortality.

## Discussion

We studied 3,180 patients diagnosed with TTS in Sweden between 2015 and 2022 from the SCAAR registry, applying a gradient-boosting machine learning algorithm to uncover significant predictors of 30-day mortality. Our study recognizes the pivotal role of the treating hospital in predicting 30-day mortality among TTS patients. Previous research mainly focused on variables centered on the patient, whereas our study identified a prominent role of the treating hospital.

The impact of hospital resources on the outcomes of TTS patients is multifaceted and significantly influenced by various factors. One critical aspect is the role of certain hospitals in admitting high-risk TTS cases transferred from smaller facilities. This, along with the number of TTS patients diagnosed and treated, the availability and implementation of coronary angiography, and access to advanced treatments, crucially affects patient prognosis. A critical aspect of advanced treatment for TTS is the timely use of mechanical circulatory support (MCS) such as Extracorporeal Membrane Oxygenation (ECMO) and Impella [23], especially for patients facing severe hemodynamic instability [24]. However, it’s important to note that these mechanical support devices are not available in all hospitals, highlighting disparities in treatment capabilities across different healthcare settings. It’s crucial to acknowledge that hospitals may vary in their proficiency and readiness to diagnose and treat not only TTS but also other severe conditions that can trigger TTS, such as stroke, internal bleeding, sepsis, trauma, respiratory insufficiency, or complications from malignancies. We have illustrated these hospital-level determinants by three real-world cases with fatal outcomes (see the supplement).

The hospitals may differ in the proficiency of healthcare teams and the accessibility of essential resources for TTS management. Consequently, efforts to improve outcomes should aim at augmenting institutional capabilities, including education and judicious distribution of resources. Specifically, the pharmacological management of TTS can vary widely and may have significant implications for patient outcomes [3, 25]. One potential area of variability is using vasoactive agents, including positive and negative inotropes and diuretics. In TTS, these treatments can be detrimental [26]. The main challenge in the pharmacological management of TTS lies in our limited understanding of the syndrome’s pathophysiology. TTS is a complex condition with multifaceted pathophysiology that is not yet fully understood. The myocardial stunning seen in TTS is thought to result from an acute surge in catecholamines in response to stress, but other factors are likely at play. Given these complexities, potent vasoactive agents in TTS should be cautiously approached until we better understand the syndrome’s underlying mechanisms. Our study identified the treating hospital as the most critical predictor of 30-day mortality in TTS patients. This underscores the need for further research to elucidate this condition’s optimal pharmacological management strategies. This could involve multicenter randomized controlled trials comparing different treatment strategies and further studies exploring the pathophysiology of TTS to guide the development of more targeted therapies [21]. Currently, there is only one study of this kind, initiated by our group and utilizing the Swedish national registry platform — BROKEN SWEDEHEART (NCT04666454) [27].

An additional aspect that merits attention is the role of timely and accurate diagnosis and adequate risk estimation in TTS patients. The ability to promptly recognize and accurately diagnose TTS and an informed and precise estimation of the risks involved can be decisive for patient outcomes. This implies that not only does proficiency in management matter but also the acuteness in the initial approach to TTS. Strengthening diagnostic capabilities and improving risk assessment protocols could be vital to improving patient outcomes. The importance of clinical presentation in angiography is consistent with existing literature, indicating that more severe presentations are associated with worse outcomes [28, 29]. Likewise, the significance of creatinine level [21] and Killip class (including different stages of cardiogenic shock [22]), both markers of disease severity, align with previous research highlighting their predictive value in heart failure syndromes [30].

This study has several limitations due to the nature of the data available. First, the registry data did not capture all potential variables that could impact the model’s predictions. Specifically, it lacks information on specific comorbidities not listed in Table 1 and data on secondary TTS, different types of TTS, ejection fraction, treatment with inotropic agents, and biomarkers, which are not reported in the SCAAR registry. The absence of these variables could limit the model’s accuracy and generalizability. Second, there was an increase in missing data during the study period, which differed between hospitals. The hospitals that document the lowest number of cases may exclude low-risk cases, whereas hospitals with the greatest number of instances report many low-risk cases. However, the imputation process allowed us to incorporate all available data, minimizing potential biases. Third, the analysis was based on data from a single country, which might limit the generalizability of our findings. However, the main goal was to provide reliable data that could lead to the generation of hypotheses for further investigation.

In conclusion, our findings highlight the importance of hospital and clinical factors in predicting 30-day mortality in TTS patients. Future research should focus on improving the management of TTS in hospitals with higher mortality rates and further refining risk stratification tools for TTS to provide personalized care.

### Electronic supplementary material

Supplementary Material 1

## Data Availability

The datasets used and/or analysed during the current study available from the corresponding author on reasonable request.
