# Supplementary material for: Importance of hospital and clinical factors for early mortality in Takotsubo syndrome: Insights from the Swedish Coronary Angiography and Angioplasty Registry
Source: BMC Cardiovasc Disord. 2024 Jul 15;24:359. doi: 10.1186/s12872-024-04023-6 (PMC11247782; doi:10.1186/s12872-024-04023-6)
Supplement: Supplementary file 1 — Supplementary Material 1 [file 12872_2024_4023_MOESM1_ESM.docx]

**Supplement**

**Importance of hospital and clinical factors for early mortality in**

**Takotsubo syndrome:**

**Insights from the Swedish Coronary Angiography and Angioplasty Registry**

Thorsteinn Gudmundsson^1^, Björn Redfors^1^, Truls Råmunddal^1^, Oskar Angerås^1^,

Petur Petursson^1^, Araz Rawshani^1^, Henrik Hagström^2^, Joakim Alfredsson^3^,

Christina Ekenbäck^4^, Loghman Henareh^5^, Kristofer Skoglund^1^, Charlotta Ljungman^1^,

Moman Mohammad^6^, Tomas Jernberg^5^, Ole Fröbert^7^,

David Erlinge^6^, Elmir Omerovic^1^

^1^ Department of Cardiology, Sahlgrenska University Hospital, Gothenburg, Sweden

^2^ Department of Cardiology, Umeå University Hospital, Umeå, Sweden

^3^ Department of Cardiology, Linköping, University Hospital, Linköping, Sweden

^4^ Department of Cardiology, Danderyd University Hospital, Stockholm, Sweden

^5^ Department of Cardiology, Karolinska University Hospital, Stockholm, Sweden

^6^ Department of Cardiology, Skåne University Hospital, Lund, Sweden

^7^ Department of Cardiology, Örebro University Hospital, Örebro, Sweden

The purpose of the patient cases described in this supplementary text is to illustrate hospital-level factors influencing clinical outcomes in TTS. The cases provide accounts of the clinical courses of patients presenting with TTS, emphasizing the impact of hospital decision-making processes on patient care and ultimate outcomes. Each case highlights specific aspects such as diagnostic oversights, procedural delays, pharmacologic choices, interdisciplinary coordination, threshold for admittance and diagnostics, and therapeutic and diagnostic approaches, shedding light on the pivotal role of hospital-level determinants in shaping TTS patient outcomes. The narratives emphasize the need for a fortified understanding of institutional capabilities and the targeted distribution of medical resources to mitigate diagnostic and therapeutic challenges, ultimately improving TTS patient care.

**Case 1—** **Critical delays and diagnostic oversights**

This account details the treatment of an eighty-year-old female patient with a history of hypertension, hypothyroidism, and hyperlipidemia. Eight years prior, she had experienced a non-ST elevation myocardial infarction, treated via percutaneous coronary intervention and stent placement in her left anterior descending artery. After presenting with significant chest pain in the evening, she sought medical attention in the emergency department of her local hospital. Given her ongoing chest discomfort and a new right bundle branch block evident on the electrocardiogram, the on-call cardiologist at a secondary medical facility was consulted. Subsequently, following established protocols, she was referred to a tertiary-care emergency department for further management.

Upon admission to the tertiary care hospital, the patient was found to have elevated troponin levels. Consequently, she was admitted to the coronary care unit, where coronary angiography was performed. The angiography results, however, revealed no plaque rupture or significant coronary artery obstructions. An investigation with contrast ventriculography discovered extensive apical akinesia. Apical akinesia was confirmed by echocardiography with an ejection fraction of 35%. When the patient spoke with the cardiac unit's supervising cardiologist the following morning, it was revealed that the patient had started experiencing diarrhea, emesis, and epigastric pain simultaneously the night before. Over the next few hours at night, anuria appeared along with generalized abdominal pain but not overt peritoneal irritation. Laboratory findings showed increased serum creatinine, lactate, and C-reactive protein concentrations. Intravenous furosemide was used to induce diuresis, and following surgical consultation, an urgent abdominal CT scan was obtained.

When the CT scan was ordered at 11:26 on the second day of the hospital stay, it revealed an occluded superior mesenteric artery and a noticeable lack of contrast perfusion throughout the small intestine, except for the duodenum. The surgical procedure, which included resection of the small intestine and embolectomy, took longer than expected to complete until later that afternoon, most likely due to imaging procedure delays and ensuing communication lags. Following surgery, the patient was sent to the critical care unit in preparation for a potential right hemicolectomy and subsequent reevaluation. Hemodynamic parameters declined overnight, and the patient was treated with increasing dobutamine and noradrenaline infusions. Sadly, the patient passed away the following day from cardiac arrest.

This narrative underscores the potential bidirectional relationship between TTS and embolic phenomena, wherein TTS may precipitate or emerge from intestinal ischemia. The 16-hour interval from hospital admission to operative care, the protracted timelines in securing and relaying CT imaging, and the incremental delays culminating in surgical consultation characterize the systemic limitations. Pharmacologic choices concerning vasopressors, inotropes, and diuretics might have exacerbated the hemodynamic fragility. Specifically, the concomitant administration of noradrenaline and dobutamine, both exerting beta1-agonist effects, may have intensified left ventricular outflow obstruction and further destabilized blood pressure—effects potentiated by injudicious diuretic use considering echocardiographic evidence of a hypovolemic, underfilled left ventricle.

Notably, the procedural narrative reflects an absence of cardiologic oversight in the perioperative period, particularly in optimizing TTS management. This case highlights the critical importance of hospital-level factors in determining patient outcomes with TTS, echoing the themes of therapeutic and diagnostic decision-making that have severe repercussions.

**Case 2—Mid-ventricular TTS with intestinal ischemia**

This case describes the clinical course of a female patient who was septuagenarian and developed exertional dyspnea and severe lethargy over a week. Her subsequent weakness and disorientation prompted her to call for an ambulance for medical assistance. An ECG performed before hospitalization revealed anterior ST-segment elevation, necessitating prompt admission to a tertiary care center. Following emergency coronary angiography, no flow-limiting lesion was seen. On the other hand, ventriculographic examination revealed mid-ventricular akinesia, which suggests a mid-ventricular TTS. On the second day of the hospital stay, the patient's clinical condition rapidly deteriorated. The systolic blood pressure dropped to 85 mmHg, and she developed atrial fibrillation with fast ventricular rate. The patient was transferred to the intensive care unit due to the possibility of septic shock, even in the absence of pyrexia or increases in C-reactive protein initially. A central venous catheter and PiCCO (Pulse Contour Cardiac Output) line were inserted to conduct invasive hemodynamic monitoring once lactate levels of 10 mmol/L were detected. The patient was treated with a broad-spectrum antibiotic regimen. Even though sinus rhythm was restored at the start of amiodarone infusions, nodal bradycardia and chronic hypotension persisted. Dopamine, isoprenaline, and noradrenaline infusions were initiated, and on the third day, continuous renal replacement therapy was started. Potential intestinal ischemia was mentioned during second-day surgeon consultations; however, a thorough investigation of this diagnosis was not carried out.

Progressive clinical deterioration led to the patient's demise on the third day of hospitalization. Post-mortem examinations revealed extensive bowel ischemia as a pivotal contributor to circulatory collapse and subsequent fatal outcome. In this instance, intestinal ischemia may have caused a septic state, a clarification not effectively distinguished during the patient's clinical course. Reflecting on the administered vasoactive pharmacotherapy—comprising isoprenaline, noradrenaline, and dobutamine—their commonality as beta1-agonists elucidates their potential to intensify left ventricular outflow tract obstruction and further diminish systemic arterial pressures. This pharmacological approach in the setting of TTS may inadvertently precipitate a cascade toward hemodynamic compromise.

The narrative in this patient case exposes the critical need for diligent and interdisciplinary investigating methods when confronted with TTS. This highlights the importance of maintaining a heightened clinical suspicion for concurrent pathologies, such as acute intestinal ischemia, that may trigger TTS. Additionally, it emphasizes the critical need for a synchronized and timely therapeutic and diagnostic approach.

**Case 3—TTS in an elderly patient with acute lymphocytic leukemia**

This case involves an eighty-three-year-old woman diagnosed with advanced acute lymphocytic leukemia (ALL). She was referred to a tertiary center hospital due to suspected ST-elevation myocardial infarction. A week prior, the treating hematologist had discussed the patient's deteriorating condition and advised continuing Imatinib (cytostatic) and cortisone while recommending against more aggressive treatments. Local palliative care was also suggested. Initially, under the administrative care of a different hospital in the county, she was transferred to the tertiary hospital for urgent coronary angiography.

Upon arrival, the patient was hemodynamically stable. It remains unclear whether the attending cardiologist or interventionalist was fully aware of the patient's ALL progression before opting for coronary angiography. Despite the uncertainties, the angiography was performed, revealing no coronary artery disease to account for the ECG changes. The ultrasound findings indicated an akinetic apical segment and hypokinetic mid-segments with hyperdynamic basal segments, consistent with TTS. Post-procedure, the patient experienced complications, including a thrombotic occlusion of the radial vein, causing a swollen right arm and a drop in hemoglobin to 78 g/L, necessitating a red blood cell transfusion. Subsequently, the patient was discharged and commenced ambulatory palliative care, passing away at home within a month. The propensity of different hospitals to opt for coronary angiography in such scenarios can significantly vary, reflecting their respective thresholds for admitting and diagnosing TTS. The existence of hospitals with differing thresholds for such admissions significantly influences the explanatory power of hospitals regarding TTS outcomes. Although it is improbable that the therapeutic complications directly caused the patient's demise, they could have contributed to the acceleration of the patient's inevitable clinical demise.
